# Can daily consumption of enriched fatty acids diet be effective in improving metabolic syndrome? An attractive paradox for walnut kernel

**DOI:** 10.1002/fsn3.3972

**Published:** 2024-01-28

**Authors:** Melika Samei, Nafiseh Dowlatkhahi, Motahareh Boozari, Hossein Hosseinzadeh

**Affiliations:** ^1^ School of Pharmacy Mashhad University of Medical Sciences Mashhad Iran; ^2^ Department of Pharmacognosy, School of Pharmacy Mashhad University of Medical Sciences Mashhad Iran; ^3^ Department of Pharmacodynamics and Toxicology, School of Pharmacy Mashhad University of Medical Sciences Mashhad Iran; ^4^ Pharmaceutical Research Center, Pharmaceutical Technology Institute Mashhad University of Medical Sciences Mashhad Iran

**Keywords:** anti‐diabetic, *Juglans regia*, metabolic syndrome, obesity, signaling pathway, walnut

## Abstract

Imagine consuming a daily diet rich in fatty acids to help treat diseases such as hypertension and obesity. This concept presents an attractive paradox. In particular, consuming walnut kernels is beneficial for treating diseases associated with metabolic syndrome (MetS), including type 2 diabetes, cardiovascular disease, dyslipidemia, and obesity. Different parts of the *Juglans regia* tree (family Juglandaceae), including its leaves, green husks, bark, and septum, have shown promising effects on pathological conditions related to MetS. The therapeutic advantages of consuming walnut kernels for MetS can be attributed to the presence of polyunsaturated fatty acids and polyphenolic compounds such as juglone and ellagic acid. Diets enriched with walnut kernel have a positive impact on MetS complications by reducing diastolic blood pressure, improving blood lipid profiles, lowering fasting blood sugar levels, and increasing insulin sensitivity. The potential cellular mechanisms responsible for these benefits involve activating the cholesterol hemostasis pathway by inhibiting sterol regulatory element‐binding proteins (SREBPs), proprotein convertase subtilisin/kexin type 9 (PCSK9), and cholesteryl ester transfer protein (CETP). Furthermore, other by‐products of walnuts, such as leaves and green husks, have also demonstrated effectiveness in managing MetS. These findings highlight the potential of incorporating walnut‐based products into our diets as a natural approach to combating MetS and its complications.

## Graphical abstract

Metabolic syndrome (MetS) is a collection of severe pathological conditions that occur together, including type 2 diabetes, cardiovascular disease, dyslipidemia, and obesity. *Juglans regia* tree (family Juglandaceae), including its leaves, green husks, bark, and septum, has shown promising biological effects on pathological conditions related to MetS. Daily enriched diets from walnut kernels have demonstrated positive effects on cardiovascular disease, obesity, diabetes, and metabolic syndrome.

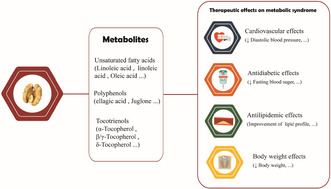

## 1 INTRODUCTION

In recent years, lifestyle changes toward a high‐calorie diet and mobility reduction have increased the risk of metabolic syndrome (MetS) (Hwang et al., 2019). According to the World Health Organization (WHO) criteria, MetS is defined as the presence of three or more of the following risk factors, including hyperinsulinemia, dyslipidemia (TG ≥150 mg/dL and HDL <40 mg/dL), hyperglycemia (FBS ≥110 mg/dL), hypertension (BP ≥140/90 mmHg), and obesity (waist‐to‐hip ratio >0.9 in men and >0.85 in women). Other conditions such as high body mass index (BMI) and albuminuria could also be introduced as risk factors for MetS. However, the National Cholesterol Education Program (NCEP) provides a slightly different definition: MetS is a metabolic perturbation that involves the coexistence of at least three conditions: (a) obesity, (b) dysglycemia, (c) cardiovascular disease, and (d) dyslipidemia (Grundy et al., 2005; Parikh & Mohan, 2012).

Some medicinal plants with therapeutic effects in MetS were known in previous studies such as *Rosmarinus officinalis* (Hassani et al., 2016), *Vitis vinifera* (Akaberi & Hosseinzadeh, 2016), *Berberis vulgaris* (Tabeshpour, Imenshahidi, et al., 2017), *Panax ginseng* (Aminifard et al., 2021), *Nigella sativa* L. (Razavi & Hosseinzadeh, 2014), *Persea americana* (Tabeshpour, Razavi, et al., 2017), *Garcinia mangostana* (Tousian Shandiz et al., 2017), *Crataegus pinnatifida* (Dehghani et al., 2019), *Portulaca oleracea* (Ebrahimian et al., 2022), *Silybum marianum* (Tajmohammadi et al., 2018), *Citrus paradisi* (Razavi & Hosseinzadeh, 2019), *Ginkgo biloba* (Eisvand et al., 2020), *Aloe vera* (Shakib et al., 2019), *Abelmoschus esculentus* (Esmaeilzadeh et al., 2020), *Capsicum annuum* (Sanati et al., 2018), *Boswellia serrata* (Mahdian et al., 2020), *Solanum melongena* (Yarmohammadi et al., 2021), and *Zingiber officinale* (Salaramoli et al., 2022).

A walnut is an edible nut from the *Juglans regia* tree (family Juglandaceae). The various parts of *J. regia* such as green husks, bark, leaves, and shells are also used in traditional medicine and the pharmaceutical industry (Almonte‐Flores et al., 2015; Boulfia et al., 2021).

Nutritionally, walnut is a rich source of unsaturated fatty acids (α‐linolenic acid, linoleic acid, oleic acid) and contains minerals (calcium, magnesium) (Domenech et al., 2019), vitamins (γ‐tocopherol, folate) (Berryman et al., 2013; Cortés et al., 2006; Fitschen et al., 2011; Joukar et al., 2017), fiber (Joukar et al., 2017), and amino acids (L‐arginine, L‐phenylalanine, leucine, alanine, glutamic acid) (Fan et al., 2022). In addition, walnuts contain polyphenols (ellagic acid, tellimagrandin I, tellimagrandin II, juglone) (Fitschen et al., 2011; Moravej et al., 2016; Shimoda et al., 2009) and phytosterols (Fitschen et al., 2011) as biologically active metabolites.

On the other hand, *J. regia* leaf extract contains secondary metabolites such as tannins (catechin, epicatechin), polyphenols (gallic acid, caffeoylquinic acid, ellagic acid, chlorogenic acid, etc.), flavonoids (juglone, quercetins), and essential oils (Hosseini, Huseini, et al., 2014; Hosseini, Jamshidi, et al., 2014; Nasiry, Khalatbary, Ahmadvand, et al., 2017). Walnuts kernel extract also contains tannins (pedunculagin), polyphenols (tellimagrandin I, tellimagrandin II, ellagic acid), polyunsaturated fatty acids, and tocotrienols (Rusu, Fizesan, et al., 2020; Rusu, Georgiu, et al., 2020; Shimoda et al., 2009).

**FIGURE 1:**
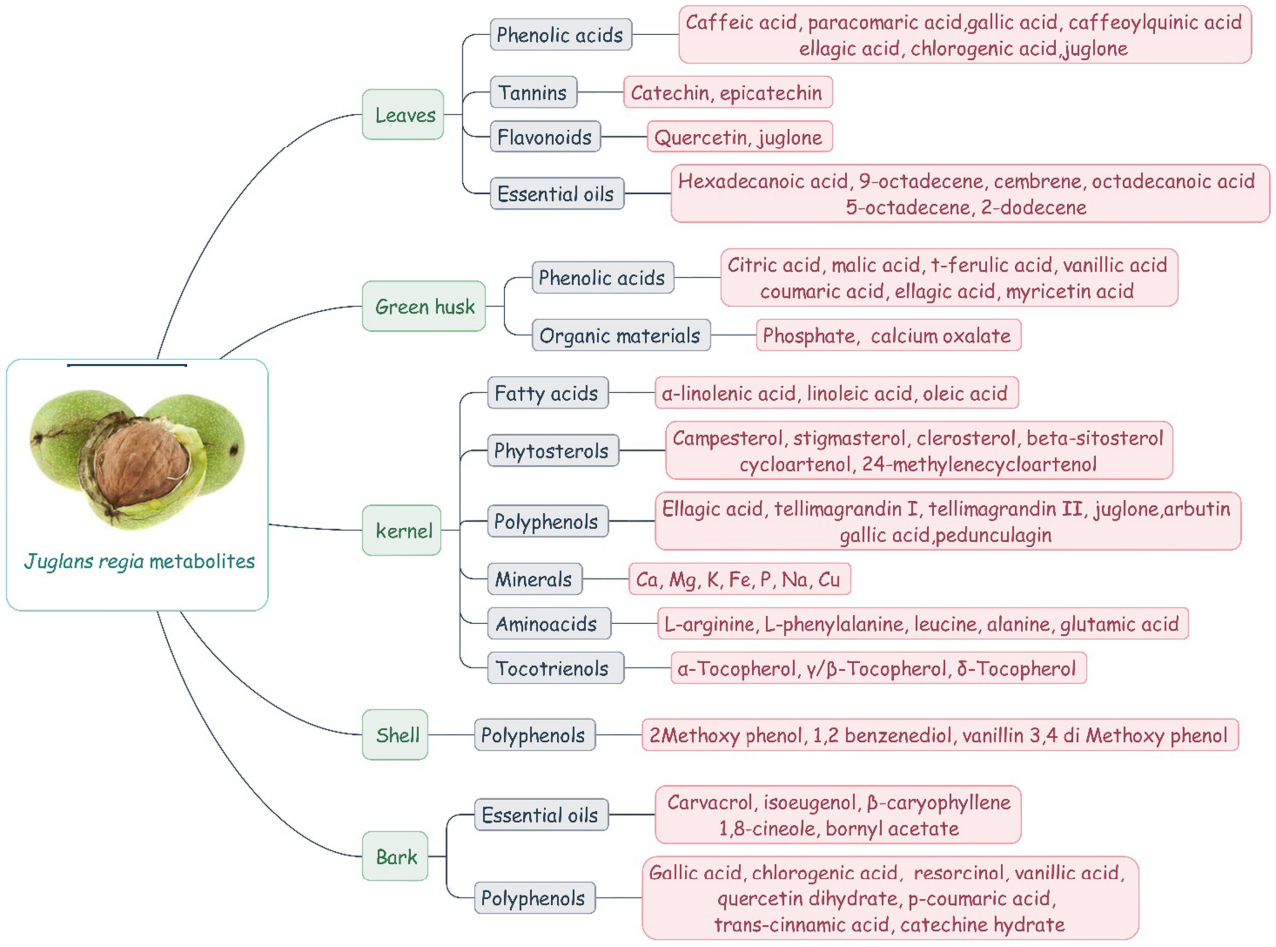
Secondary metabolites from the different parts of *Juglans regia*.

Furthermore, polyphenols such as vanillic acid, t‐ferulic acid, myricetin acid, ellagic acid, and coumaric acid have been extracted from green husk (Hosseini, Huseini, et al., 2014; Hosseini, Jamshidi, et al., 2014; Wang et al., 2019). Secondary metabolites from various parts of *J. regia* are summarized in Figure 1. In this article, we have studied the effects of walnuts in treating metabolic syndrome and related risk factors.

**FIGURE 2:**
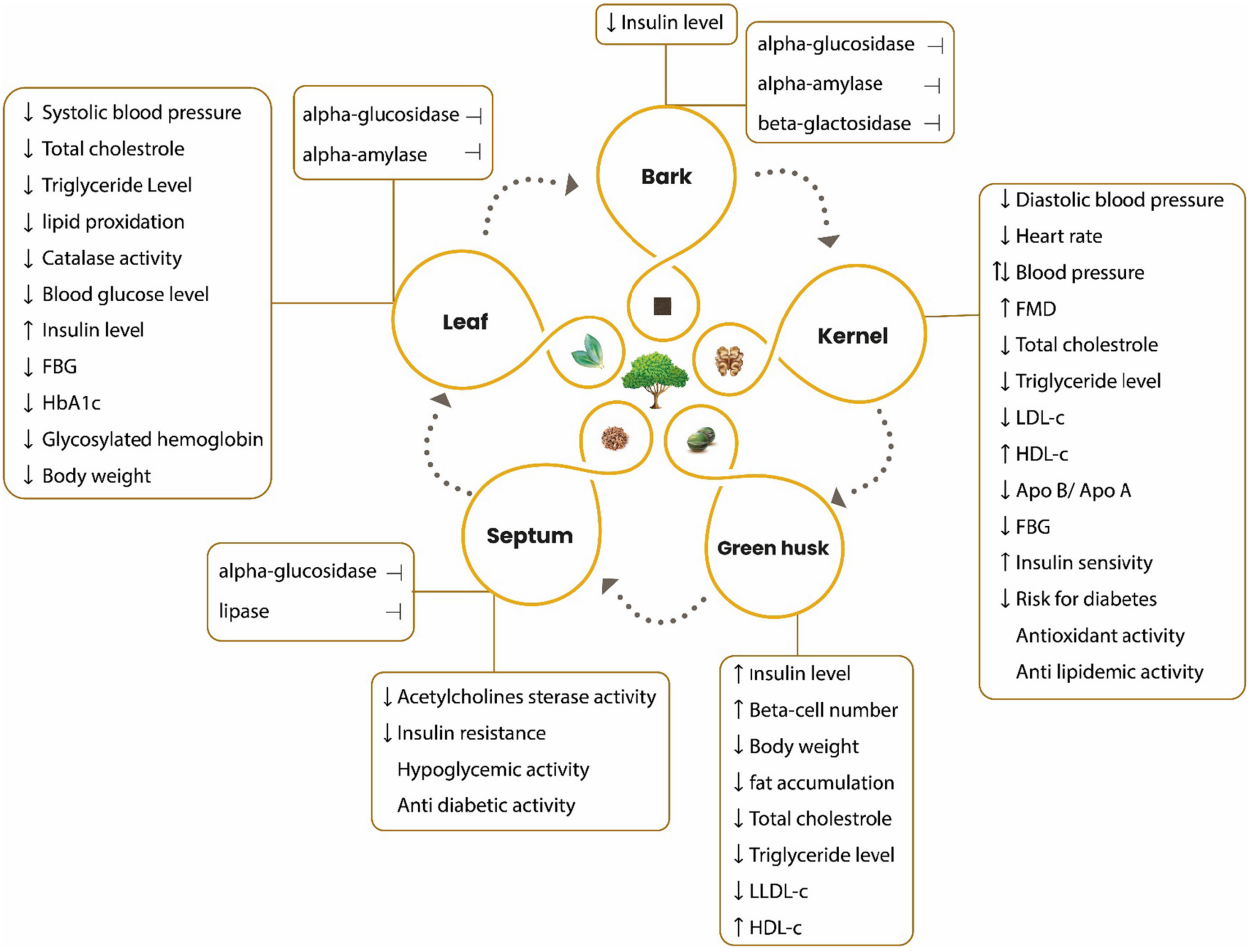
Biological effects of different parts of *Juglans regia* on pathological conditions related to metabolic syndrome.

It seems that most research has emphasized on the nutritional value of walnut kernels in reducing the risk of MetS, while the potential effects of other parts of *J. regia* on MetS have not been extensively studied. Various parts of the plant, including the kernel, leaf extract, green husk extract, kernel and septum extract, and even the bark of the tree, are effective in treating complications associated with metabolic syndrome. Different studies have highlighted the promising biological effects of different parts of *J. regia* on pathological conditions related to metabolic syndrome, as summarized in Figure 2.

## 2 METHODS

For this review, articles on the topic published through July 2022 were searched in various databases or search engines, including PubMed, Scopus, Google Scholar, and Web of Science. The search was performed using the following keywords: “*Juglans regia*”, “Walnut”, “hypertriglyceridemia”, “dyslipidemia”, “high cholesterol”, “high triglyceride”, “hyperlipidemia”, “atherogenic”, “atherosclerosis”, “hypertension”, “blood pressure”, “diuretic”, “antihypertensive”, “hypertensive”, “hypotensive”, “Insulin”, “anti‐hyperglycemia”, “diabetes”, “hypoglycemic”, “blood glucose”, “antidiabetic”, “antihyperglycemic”, “metabolic syndrome”, “food intake”, “bodyweight”, “obesity”, and “anti‐obesity”. All related English articles addressing the effects of *J. regia* on metabolic syndrome were included in the review process.

## 3 CARDIOVASCULAR DISEASE

Cardiovascular diseases (CVD) are a general medical term that encompasses all diseases of the blood circulatory system, including congenital heart defects (CHD), heart failure (HF), hypertension (HTN), and stroke. HTN is a medical condition that is considered a major risk factor for cardiovascular diseases (Tsao et al., 2022). Atherosclerosis is a serious condition where arteries become thickened or hardened with fatty plaques and is associated with stroke, coronary artery disease, and peripheral vascular disease (Daugherty et al., 2017). According to studies, natural products play a critical role in the prevention and treatment of cardiovascular disorders (Hernández et al., 2023; Shukla et al., 2010).

The biological effects of walnut kernels on cardiovascular diseases, especially atherosclerosis are summarized in Table 1.

**TABLE 1:** The biological effects of the walnut kernel on cardiovascular disease.

	Design of study	Part of plant	Dose	Finding	Reference
Clinical studies	Clinical trial *N* = 3341 Observation study	Walnut kernel		↓Diastolic BP (especially in young to middle‐aged adults) ↓Heart rate Without effect on SBP	Steffen et al. (2021)
	Clinical trial *N* = 1833	Walnut kernel	How many times a week	↓Risk of cardiovascular disease in the population at high CVD risk	Guasch‐Ferré et al. (2021)
	Clinical trial *N* = 236 Elder participants	Walnut kernel	15% energy	↓SBP in mild hypertension (especially in elderly patients) Better BP regulation	Domenech et al. (2019)
	Clinical trial *N* = 100 A standard diet with reduced energy density or an energy‐reduced diet enriched with walnuts (15% of energy)	Walnut kernel		Maintain ↓ systolic blood pressure for 6 months ↓Diastolic blood pressure	Rock et al. (2017)
	Clinical trial *N* = 127	Walnut kernel	30 g/day	↓Progress of atherosclerotic lesions (since childhood) Anti‐atherosclerosis activity	Regulska‐Ilow et al. (2012)
	Clinical trial *N* = 112 Diet included/excluded walnuts	Walnut kernel	56 g	↓Cardiometabolic risk factors	Njike et al. (2019)
	Clinical trial *N* = 100	Walnut kernel	15 cc/day	↓Risk for CAD	Zibaeenezhad et al. (2017)
	Clinical trial *N* = 62 Control diet, a diet with walnuts, a diet with cashew nuts	Walnut kernel	20% energy	A diet rich in walnuts: ↓Baroreflex sensitivity	Schutte et al. (2006)
	Clinical trial *N* = 50 A diet with or without nuts	Raw nuts (7.5 g hazelnuts, 15 g walnuts and 7.5 g almonds)	30 g/day	Changes in median plasma IL‐6 among inflammatory markers	Casas‐Agustench et al. (2011)
	Clinical trial *N* = 46 Walnut‐enriched diet and diet without walnut	Walnut	56 g	↑Flow‐mediated vasodilation (FMD) Benefits for ↓ systolic blood pressure ↑Endothelial function in obese adults with visceral fat	Katz et al. (2012)
	Clinical trial *N* = 42 A diet that replaces SFAs with walnuts/vegetable oil	Walnut	57–99 g/day	Gut microbiota may be effective in the cardiovascular benefits of walnut kernel consumption.	Tindall, McLimans, et al. (2019)
	Clinical trial *N* = 36 Replacing saturated fat with walnuts	Walnut		The treatment effect for central diastolic blood pressure Cardiovascular benefits Significantly reduction in brachial and central mean arterial pressure	Tindall, McLimans, et al. (2019) and Tindall, Petersen, et al. (2019)
	Clinical trial *N* = 29 The effects of eating the English versus the black walnut	English walnut/Black walnut	30 g/day	Black walnut: Improvement in endothelial function	Fitschen et al. (2011)
	Clinical trial *N* = 25 Use of the meat product with or without walnuts	Walnut	136 g/week	Restructured meat products with added walnuts are considered functional foods for high‐risk CVD individuals	Olmedilla‐Alonso et al. (2008)
	Clinical trial *N* = 24 Addition of walnut oil/olive oil to the meal	Walnuts (oil)	40 g shelled walnuts	↑FMD Protection from the phenotype of endothelial cells	Cortés et al. (2006)
	Clinical trial *N* = 24	Walnut	56 g/day	Endothelial function improved	Ma et al. (2010)
	Clinical trial *N* = 12 Average American diet, alpha‐linolenic acid diet, linoleic acid diet Varies in the amount of linoleic acid and alpha‐linolenic acid	ALA (walnuts, walnut oil, and flax oil)		↓Diastolic blood pressure ↓Total peripheral resistance ↑FMD ↑Arginine‐vasopressin (AVP) +Endothelin‐1 was unchanged +Cardioprotective effects	West et al. (2010)
	Clinical trial *N* = 10	Walnut	48 g/day	↓Deleterious sphingomyelin, ceramides, and hexosylceramides are associated with cardiometabolic risk	Tuccinardi et al. (2019)
	Clinical trial *N* = 708	Walnut kernel	1, 1.5, or 2 oz. or ~ 15% of energy	↓BP	Al Abdrabalnabi et al. (2020)
	Clinical trial *N* = 112 Diet with or without walnut	Walnut kernel	56 g/day	No effects on blood pressure	Njike et al. (2015)
	Clinical trial *N* = 68	Walnut kernel		No significant effects on clotting factors activity such as von Willebrand factor (vWF), plasminogen activator inhibitor 1 (PAI‐1), factor VII, thrombin‐activatable fibrinolysis inhibitor (TAFI), and tissue plasminogen activator	Pieters et al. (2005)
In vitro & in vivo studies	In vivo Apoe−/− mice + The effect of walnuts on the amount of coagulation has not been shown	Walnut		↓Atherosclerotic plaque development in 55% of the aortic arch ↓Plaque enrichment for CD36 No change in platelet activation and thrombus formation in the bloodstream ↓Accumulation of lipids ↑Plasma antioxidant capacity ↓Prothrombin	Nergiz‐Ünal et al. (2013)
	In vivo hyperlipidemic diet with increased total walnuts/alpha‐tocopherol concentration and dietary supplements containing walnut oil/pure γ‐tocopherol	Walnut	61–150 g/kg	↓Aortic cholesterol ester concentration (as a marker for aortic atherosclerosis) ↓Aortic endothelin 1 mRNA	Davis et al. (2006)
	In vivo	Walnut		↑Blood melatonin levels correlate with the increase the antioxidant activity	Reiter et al. (2005)
	In vivo	Walnut		METS‐TG (a mouse model of metabolic syndrome): mean arterial blood pressure ↓	Scott et al. (2017)
	In vivo (dexamethasone‐induced hypertension)	Walnut kernel extract	100 and 200 mg/kg/day	Walnut extract decreased dexamethasone‐induced hypertension (comparable with captopril) Balance the redox system and NO production	Joukar et al. (2017)
	In vivo The control group and fructose‐fed group (after 9 weeks, half of the control and fructose‐fed rats received walnuts in the diet)	Walnut	2.4 g/daily	Protection against cardiovascular events	Boskovic et al. (2021)
	In vivo Isoproterenol‐induced myocardial infarction	Walnut kernels extract	300 mg/kg	Significantly protection against ISO‐induced MI	Sun et al. (2019)
	In vivo/in vitro	Walnut kernel	2.4 g	Moderately positive vasodilatory effect in healthy rats ↑Phosphorylated Akt aortic level, ↓LTCC alpha‐level, ↑SUR2B subunit of KATP level	Stanisic et al. (2021)
	In vitro/in vivo In vitro inhibitory activity of Angiotensin‐I‐converting enzyme/in vivo antihypertensive effect	Walnut protein, Walnut protein hydrolysate		WP/WPH: ↓Blood pressure effects	Liu et al. (2021)

### 3.1 Effects of walnut kernel enrich diet on CVD

#### 3.1.1 Clinical studies

Several clinical studies have been conducted to investigate the effect of consuming walnut kernels on reducing the risk of cardiovascular diseases. In these studies, the blood pressure of walnut consumers has been compared to non‐consumers. Although walnut consumption demonstrates the effects on blood pressure and heart rate, these effects were not found to be significant in many cases.

On the other hand, recent studies have shown that replacing polyunsaturated fatty acids (n‐6 PUFA or trans fatty acids) with n‐3 PUFA (α‐linolenic acid [ALA]) in dietary intake improves cardiovascular health. Therefore, consuming walnuts as a source of n‐3 PUFA could be considered a promising approach to reducing the occurrence of CVD (Tsao et al., 2022). Furthermore, Tindall and coworkers in a randomized controlled trial with 45 individuals demonstrated that replacing saturated fat with walnuts (57–99 g/day) or a‐linolenic acid, improved central diastolic blood pressure in adults at risk for cardiovascular disease (*p* = .04). This effect is not substantial but it seems that a small change in dietary diet patterns could be effective in the prevention of CVD (Tindall, McLimans, et al., 2019).

In addition, Guasch‐Ferré et al. (2021) demonstrated that walnut consumption and plasma metabolites such as lipids, amino acids, purines, and acylcarnitines could decrease the risk of CVD in the Mediterranean population. Overall, there was a lack of evidence suggesting that increasing long‐chain omega‐3 or alpha‐linolenic acid had any impact on serious adverse events, adiposity, or lipids. However, it was observed that long‐chain omega‐3 did have a slight effect in reducing triglycerides and increasing HDL levels (Abdelhamid et al., 2020).

In another study, a walnut diet led to a reduction in SBP (approximately 6 mmHg reduction, *p* = .034) and improved blood pressure regulation in elderly individuals with mild hypertension (Domenech et al., 2019).

In an observational study involving 3341 participants, coronary artery risks were compared between walnut consumers and non‐walnut consumers. It was found that the quality of the diet was higher in walnut consumers. Furthermore, daily walnut ingestion resulted in a significant reduction in DBP and heart rate (*p* = .09), but it did not affect systolic function (Steffen et al., 2021).

A large elderly cohort study conducted over 2 years with 708 participants showed that daily walnut consumption had no effect on MetS and its complications, and even the reduction in SBP was not significant (Al Abdrabalnabi et al., 2020).

Furthermore, another randomized controlled study with 112 participants revealed that a walnut‐enriched diet (56 g) for 6 months had no significant effect on blood pressure but could improve endothelial function (Njike et al., 2015).

#### 3.1.2 Effect on endothelial function

In a clinical study involving overweight adults, a daily intake of walnuts (56 g) improved endothelial function and significantly increased flow‐mediated dilation (*p* = .019) without significant weight change (Katz et al., 2012). Furthermore, in a randomized controlled crossover trial, a walnut‐enriched diet (56 g/day) in 24 diabetic patients (mean age 58 years) significantly improved endothelium‐dependent vasodilatation with flow‐mediated dilation (FMD) increased by 25% (*p* = .04) (Ma et al., 2010).

In another randomized crossover study, it was determined that the addition of walnut (40 g) or olive oil (25 g) acutely improved FMD and endothelial function in hypercholesterolemic patients (Cortés et al., 2006). The polyunsaturated fatty acids (PUFA) present in walnuts decreased DBS, increased FMD (+34%), and improved cardiovascular responses to stress compared to the average American diet (West et al., 2010).

### 3.2 Mechanism of protection in CVD

An in vivo study on isoproterenol‐induced myocardial infarction revealed that walnut kernels have protective effects on MI rats, by decreasing hydroperoxide and thiobarbituric acid reactive substances (TBARS) levels in the heart compared with ISO‐control rats (*p* < .05). Additionally, walnut kernels increased antioxidant enzyme activities (CAT, SOD) compared with ISO rats induced alone (Sun et al., 2019). Another study carried out in rats showed the normalization of hypertension by balancing the redox system and the production of NO (Joukar et al., 2017).

The predicted mechanisms for the therapeutic effect of walnut consumption on CVD risk reduction may be associated with a reduction in endothelin‐1 mRNA in the aorta and cholesterol ester concentration in the aorta (Davis et al., 2006). Nergiz‐Ünal et al. (2013) also reported a 55% reduction in the progression of atherosclerotic plaques in the aortic arch by reducing lipid and prothrombin accumulation.

Overall, research suggests that consuming walnuts may have promising effects in reducing the risk of cardiovascular disease. The majority of research has focused on the impact of a walnut‐enriched diet concerning CVD, while the potential effects of other parts of *J. regia* on CVD have not been extensively studied. However, it is still unclear whether these benefits are related to PUFA or secondary metabolite.

Generally, there have been conflicting results from randomized trials regarding the effect of a walnut kernel‐enrich diet on CVD. While some studies have shown that walnut‐enriched diets (56 g/day) can significantly lower diastolic blood pressure (about 2–3 mmHg reduction), a cohort study found no significant effect on blood pressure in elderly individuals. Clinical studies have not shown a significant effect on vessel stiffness, but several studies have demonstrated the therapeutic effects of a walnut‐enriched diet on endothelial function. Based on in vitro and in vivo studies, potential mechanisms for protection from CVD include antioxidant activity, regulation of NO production, and a reduction in endothelin‐1 mRNA expression.

## 4 HYPERLIPIDEMIA

Dyslipidemia is a disease in which the lipid profile changes when the total cholesterol is ≥240 mg/dL or HDL‐C is <40 or the LDL‐C is ≥130. One of the major risk factors for the progression of atherosclerosis is cholesterol. Based on U.S. healthcare data, hyperlipidemia was the 35th most expensive disease in 2016 (Tsao et al., 2022). The biological effects of walnut consumption on dyslipidemia are summarized in Table 2.

**TABLE 2:** Walnut consumption effects on hyperlipidemia.

	Design of study	Part of plant	Dose	Finding	Reference
Clinical studies	Clinical trial *N* = 9660	Nuts (hazelnuts, walnuts, pistachios and almonds)	Mean frequency nut consumption = 2.4 ± 2.1 per week	↓Total cholesterol ↓LDL ↓Triglyceride (TG) ↓Apo B/apo A ratio Frequent nut consumption (4 ≤ times per week) had a significant reverse effect on risk factors of dyslipidemia in all four models except for high LDL and low apo A	Askari et al. (2013)
	Clinical trial *N* = 1265	Walnut	0.88–5.68 servings/week	↓Risk of MetS (each additional week of walnuts consumption reduced the incidence of MetS by 3%)	Hosseinpour‐Niazi et al. (2017)
	Clinical trial *N* = 330	Walnut	15% of energy (~30–60 g/day) for 1 year	↓LDL‐cholesterol (LDL‐C) by influencing the expression of circulating microRNAs (c‐miRNA)	Gil‐Zamorano et al. (2022)
	Clinical trial *N* = 245 Overweight and obese women	Walnut	18% energy	↓Triglycerides ↓Total cholesterol ↑HDL (compared with other diets) ↓LDL in insulin‐sensitive women +Favorable changes in lipid levels	Le et al. (2016)
	Clinical trial *N* = 112 A diet with or without walnut	Walnut	56 g/day	↓Total cholesterol ↓LDL	Njike et al. (2015)
	Clinical trial *N* = 100	Walnut	15 cc/day	↓Total cholesterol levels, ↓triglyceride level, ↓LDL level, ↑HDL level, ↓total cholesterol to HDL ratio	Zibaeenezhad et al. (2017)
	Clinical trial *N* = 100 A standard diet with reduced energy density or an energy‐reduced diet enriched with walnuts (15% of energy)	Walnut	15% of energy	↓Total cholesterol, ↓LDL, ↓Triglyceride level	Rock et al. (2017)
	Clinical trial *N* = 90 Through bioactive phytochemicals that separate the two halves of the walnut kernel	Walnut kernel	25 g/day, 50 g/day	↓Total cholesterol levels ↓LDL‐c ↓Triglyceride levels ↑HDL‐c	Shazia Ashraf et al. (2020)
	Clinical trial *N* = 84	Walnut	45 g/day	↑HDL‐C Concentration	Hwang et al. (2019)
	Clinical trial *N* = 60	Walnut	3 g/day	↓Plasma TG concentrations	Zibaeenezhad et al. (2003)
	Clinical trial *N* = 58	Walnut	30 g/day	↑Ratio of HDL/total cholesterol ↑HDL ↓LDL (10%)	Tapsell et al. (2004)
	Clinical trial *N* = 49 Walnuts replaced with monounsaturated fat	Walnut	35% of the energy	↓Total cholesterol level ↓LDL cholesterol level ↓Lipoprotein(a) level (only in men)	Zambón et al. (2000)
	Clinical trial *N* = 40 Replacing walnut in reference diet	Walnut	52 g/10 mJ of diet formula/day	↓Total cholesterol concentration ↓LDL cholesterol concentrations ↓The ratio of low‐density lipoprotein to high‐density lipoprotein ↓Apolipoprotein B concentration +Favorably modifies the lipoprotein profile	Iwamoto et al. (2000)
	Clinical trial *N* = 36 Replacing saturated fat with walnuts	Walnut		↓Total cholesterol ↓HDL‐c ↓LDL‐c ↓Non‐ HDL cholesterol	Tindall, McLimans, et al. (2019) and Tindall, Petersen, et al. (2019)
	Clinical trial *N* = 36	Walnut	31 g/day	↓Low‐density lipoprotein‐cholesterol ↓Apoprotein B	Kalgaonkar et al. (2011)
	Clinical trial *N* = 29 The effects of English versus Black walnut consumption	English walnut/black walnut	30 g/day	English walnut: ↓Total cholesterol ↓LDL levels ↑HDL in men Improved blood lipids ↑RBC polyunsaturated fatty acids Black walnut: ↑Total cholesterol, LDL level, and HDL level, in women ↓Total cholesterol, LDL level, and HDL level, in men	Fitschen et al. (2011)
	Clinical trial *N* = 25 Use of the meat product with or without walnuts	Walnut		↓Total cholesterol ↓LDL cholesterol	Olmedilla‐Alonso et al. (2008)
	Clinical trial *N* = 23 Diet + walnut	Walnut		↓Total cholesterol concentrations ↓LDL‐c concentrations (the walnut supplement lowered the cholesterol preferentially in the small LDL) ↓HDL‐cholesterol concentrations (The decrease was observed mainly in the large HDL particles)	Almario et al. (2001)
	Clinical trial *N* = 18 In men Replace part of the reference diet with walnut	Walnut	20% of the calories	↓Serum levels of total cholesterol ↓HDL‐c ↓LDL‐c +favorably modifies the lipoprotein profile in normal men	Sabaté et al. (1993)
	Clinical trial *N* = 15	Walnut	Whole walnuts (85 g), separated nut shells (5.6 g), defatted nut meat (34 g) and nut oil (51 g)	Positive effects of whole walnuts on reverse cholesterol transport (Improvement in cardiovascular risk through cholesterol‐lowering effects)	Berryman et al. (2013)
	Clinical trial *N* = 15	Walnut	48 g/day	↑Apolipoprotein A concentration	Aronis et al. (2012)
	Clinical trial *N* = 10 In men with polygenic hypercholesterolemia Walnuts replaced with unsaturated fat	Walnut	35% of total energy	↓Serum total cholesterol ↓LDL cholesterol ↓Apolipoprotein B level	Muñoz et al. (2001)
	Clinical trial *N* = 10	Walnut	48 g/day	↓Fasting small and dense LDL particles ↑Postprandial large HDL particles	Tuccinardi et al. (2019)
	Clinical trial (placebo group and intervention group)	7 g of boiled walnut as a snack	7 g	↓LDL‐ c levels within 42.1% (related to ↓PCSK9 and ↓APOE activities) ↑HDL levels by 33.6% (related to ↓CETP activities)	Amadi et al. (2022)
In vitro & in vivo studies	In vivo	Walnut kernel extract	50, 100, and 200 mg/kg	+↓Triglyceride activity by improving beta‐oxidation of peroxisomal fatty acids in the liver	Shimoda et al. (2009)
	In vivo A diet containing whole walnuts/elevated fat/low‐fat	Walnut	155 g	↓LDL	Davis et al. (2012)
	In vivo Transgenic adenocarcinoma of the mouse prostate (TRAMP) PCa model	Walnut oil/whole walnut	100 g/kg	↓Cholesterol	Kim et al. (2014)
	In vivo	Walnut		METS‐TG (a mouse model of MetS): ↑Plasma cholesterol ↑Triglyceride levels WT (wild‐type mice): ↓Cholesterol No effect on triglycerides METS‐TG and WT: ↑Expression of genes in the liver associated with inflammation, fibrosis, cellular stress, and metabolism	Scott et al. (2017)
	In vivo Injection of streptozotocin (40 mg/kg) on days 6, 7 and 8 of pregnancy	Walnut (oil)	Appropriate dose: 900 mg/kg	Mitigate abnormal changes in lipid profiles in plasma and liver tissue ↓Oxidative stress	Sun et al. (2020)
	In vivo Isoproterenol (ISO)‐induced myocardial infarction (MI)	Walnut kernels extract	300 mg/kg	The walnut kernel has anti‐lipid, peroxidative, antioxidant	Sun et al. (2019)
	In vivo	Walnut		↓Triglyceride levels ↓Cholesterol levels	Nergiz‐Ünal et al. (2013)
	Ex vivo	Walnut oil	Phytosterols: 10 μg/mL TPC: 12.5 × 10–3 μg/mL	↓Total cholesterol and triacylglycerol by Phytosterols and TPC (total phenolic content)	Gao et al. (2022)

### 4.1 Effects of walnut kernel enriched diet on hyperlipidemia

In a clinical study with 9660 participants, Askari et al. (2013) demonstrated that frequent consumption of edible nuts (pistachios, walnuts, hazelnuts, and almonds) particularly ≥4 times a week was significantly associated with a lowering in the level of total cholesterol, triglycerides, LDL‐c, and apo lipoprotein B/apo lipoprotein A ratios (*p* < .05).

In the Korean randomized, controlled crossover study involving 84 patients with MetS, it was observed that daily consumption of walnuts (45 g) for 16 weeks could increase HDL‐C (*p* = .028) (Hwang et al., 2019). In another clinical study with 245 participants, significant changes in lipid profile were observed. In this study, the level of HDL was increased in comparison to a low‐fat diet group (*p* = .05) and a low‐carbohydrate diet group (*p* < .01) (Le et al., 2016). The consumption of a walnuts enriched diet (30 g/day) by 58 patients caused a significant increase in HDL to total cholesterol ratio (*p* = .049) and HDL levels (*p* = .046). Additionally, there was a 10% decrease in LDL‐C levels (Tapsell et al., 2004). In another clinical study with 49 patients, walnut consumption significantly reduced total cholesterol (4.1%), LDL (5.9%), and lipoprotein levels (6.2%) (Zambón et al., 2000).

A clinical study by Kalgaonkar et al. (2011) on walnut consumption revealed an 11% decrease in apolipoprotein B and Aronis et al. (2012) demonstrated a significant increase in apolipoprotein A (*p* = .03). Additionally, a recent meta‐analysis of randomized controlled trials showed an improvement in lipid profile, especially in obese/overweight patients (TC: −8.58 mg/dL, LDL: −5.68 mg/dL, TG: −10.94 mg/dL) (Alshahrani et al., 2022).

### 4.2 Mechanism of anti‐hyperlipidemic activity

Another study in participants with polygenic hypercholesterolemia concluded that the mechanism of LDL‐c reduction was a 50% increase in LDL‐c uptake by HepG2 cells (Muñoz et al., 2001).

An in vivo study conducted in rats with streptozotocin‐induced gestational diabetes supported the reduction of the unusual lipid profile by walnut consumption via suppression of relative mRNA expression of sterol regulatory element‐binding transcription factor 1, acetyl‐coenzyme A, carboxylase fatty acid synthase, and stearoyl‐CoA desaturase 1 (Sun et al., 2020). Another study denotes a 36% reduction in total cholesterol and a 23% reduction in triglyceride compared to the control group (Nergiz‐Ünal et al., 2013).

“In participants with polygenic hypercholesterolemia, another study concluded that the mechanism of LDL‐c reduction was a 50% increase in LDL‐c uptake by HepG2 cells (Muñoz et al., 2001). An in vivo study conducted in rats with streptozotocin‐induced gestational diabetes supported the reduction of an abnormal lipid profile by walnut consumption, achieved through the suppression of relative mRNA expression of sterol regulatory element‐binding transcription factor 1, acetyl‐coenzyme A, carboxylase fatty acid synthase, and stearoyl‐CoA desaturase 1 (Sun et al., 2020). Another study denotes a 36% reduction in total cholesterol and a 23% reduction in triglycerides compared to the control group (Nergiz‐Ünal et al., 2013).”

An in vivo study with walnut kernels in isoproterenol‐induced myocardial infarction showed the normalization of myocardial tissue lipids (Sun et al., 2019). Additionally, the consumption of kernel extract indicated a significant decrease in triglycerides and an increase in the expression of hepatic peroxisome proliferator‐activated receptor alpha (PPAR α) and acyl‐COA oxidase 1 (ACOX_1_) (Shimoda et al., 2009).

According to literature, daily walnut kernel consumption can decrease the levels of total cholesterol, LDL, and triglycerides while increasing the level of HDL. The possible mechanism of these effects is the suppression of relative mRNA expression of SREBPs and an increase in the expression of PPAR α ACOX_1_.

## 5 DIABETES

Diabetes is a group of diseases that disrupt glucose regulation (Tsao et al., 2022). According to the WHO report, the number of diabetic people has increased from 108 million in 1980 to 422 million in 2014, with a higher prevalence in low‐ and middle‐income countries. In the United States alone, diabetes was the leading cause of death for 87,647 people in 2019 (Tsao et al., 2022). The biological effects of walnut consumption on diabetes are summarized in Table 3.

**TABLE 3:** Walnut consumption effects on diabetes.

	Design of study	Part of plant	Dose	Finding	Reference
Clinical studies	Clinical trial *N* = 34,121 NHANES	Walnut		↓Risk for diabetes	Arab et al. (2018)
	Clinical trial *N* = 1833	Walnut		↓Risk of incident T2D in women	Guasch‐Ferré et al. (2021)
	Clinical trial *N* = 112 Diet included/excluded walnuts	Walnut	56 g	↓Risk of developing T2DM	Njike et al. (2019)
	Clinical trial *N* = 84	Walnut	45 g/day	↓Fasting glucose level	Hwang et al. (2019)
	Clinical trial *N* = 50 A diet with or without nuts	Raw nuts (7.5 g hazelnuts, 15 g walnuts, and 7.5 g almonds)	30 g/day	↓Lipid responsiveness ↑Insulin sensitivity ↓Fasting insulin ↓HOMA‐insulin resistance	Casas‐Agustench et al. (2011)
	Clinical trial *N* = 50	Walnut	30 g/day	↓Fasting insulin levels	Tapsell et al. (2009)
	Clinical trial *N* = 31	Walnut	31 g/day	Insulin response↑ during OGTT (oral glucose tolerance tests), adiponectin↑, HgBA1↓	Kalgaonkar et al. (2011)
	Clinical trial *N* = 10	Walnut		↓Lipoprotein insulin resistance score ↓Glucose and the insulin range Consuming walnuts significantly ↑10 N‐glycans	Tuccinardi et al. (2019)
	Clinical trial *N* = 112 Diet with or without walnut	Walnut	56 g/day	No effects on blood glucose	Njike et al. (2015)
	Clinical trial *N* = 24	Walnut	56 g/day	No significant effect on blood glucose No significant effect on insulin sensitivity	Ma et al. (2010)
	Clinical trial *N* = 8	Walnut	250 mL/twice a day	Walnuts can control blood sugar levels in diabetics, but they may be associated with minor and major side effects	Moravej et al. (2016)
	Clinical trial *N* = 6	Walnut	400 kJ/portion	Without significant changes in glucose and insulin levels over 60 min	Megdal et al. (2010)
In vitro & in vivo studies	In vivo	Walnut		METS‐TG (a mouse model of MetS): ↓The AUC of the oral glucose WT (wild‐type mice): no effect on gAUC and MAP	Scott et al. (2017)
	In vivo Injection of streptozotocin (40 mg/kg) on days 6, 7 and 8 of pregnancy	Walnut (oil)	Appropriate dose: 900 mg/kg	↓Gestational diabetes mellitus (GDM) ↓Fasting blood glucose ↑Plasma insulin ↑Levels of hepatic glycogen	Sun et al. (2020)
	In vivo Alloxan‐induced diabetic rats	Walnut	21.3 g/42.6 g/85.2 g	↓Fasting blood glucose levels ↑Hemoglobin concentration ↓Urine output Walnut has an antihyperglycemic effect in diabetic rats	Onwuli et al. (2014)
	In vitro/in vivo	Walnut kernel	2.4 g	Tendency to increase blood insulin concentration	Stanisic et al. (2021)
	In vivo Streptozotocin‐induced diabetes	African walnut	200 mg/kg	↓FBS ↓Oxidative stress parameters malondialdehyde (MDA) level Reduction in GSH, catalase, and SOD depletion Protection against diabetes‐induced liver and kidney damage	Ajayi et al. (2022)
	Ex vivo	Walnut oil	Phytosterols: 10 μg/mL TPC: 12.5 × 10–3 μg/mL	↓Total cholesterol and triacylglycerol by phytosterols and TPC	Gao et al. (2022)

### 5.1 Effects of walnut kernel enrich diet on diabetes

#### 5.1.1 Clinical studies

A clinical study of 34,121 participants conducted by Arab et al. (2018) found that walnut consumption reduced the relative risk of diabetes by 68% compared to non‐walnut consumers. Another study involving 1833 participants demonstrated that walnuts can significantly reduce the risk of diabetes in the CVD population (*p* = .02) (Guasch‐Ferré et al., 2021). Additionally, a clinical study on walnut consumption showed a greater decrease in fasting insulin levels compared to the control group (*p* = .046) (Tapsell et al., 2009). Kalgaonkar et al. (2011) reported a significant reduction of HbA1C compared to the control group (*p* = .0006).

Similarly, clinical studies involving walnut kernels revealed a significant decrease in fasting blood glucose levels and HbA1c compared to the control group (*p* = .013, *p* = .021) (Hwang et al., 2019).

A randomized controlled study with 112 participants revealed that a walnut‐enriched diet (56 g) had no significant effect on BP but could improve the blood glucose level (Njike et al., 2015). Furthermore, another randomized clinical trial with 24 individuals demonstrated that a walnut‐enriched diet had not significant effect on insulin sensitivity (Ma et al., 2010).

### 5.2 Mechanism of anti‐diabetic activity

A study on walnut consumption in rats with gestational diabetes showed a decrease in FBG, gestational diabetes mellitus, and an increase in insulin and hepatic glycogen (Sun et al., 2020).

Based on previous studies, daily consumption of walnuts‐enriched dairy products could decrease FBS and HbA1c levels while increasing insulin levels and insulin response.

## 6 BODY WEIGHT

According to the latest guidelines, obesity is defined as a waist circumference ≥35 inches (88 cm) in women and ≥40 inches (102 cm) in men. Due to lifestyle changes, the prevalence of obesity in individuals aged 20 and older is now 39.9% in men and 41.1% in women. Studies indicate that overweighting is an important risk factor for CVD and increases the risk of MetS complications such as hypertension, diabetes, and dyslipidemia (Tsao et al., 2022). Walnut kernel administration in aging mice could significantly decrease body weight and modulate antioxidant enzyme activity (Liu et al., 2019). Therefore, the management of obesity is crucial, and one approach involves the use of anti‐obesity medications; however, it's important to note that these medications may cause side effects (Hosseini & Hosseinzadeh, 2015). The biological effects of walnut consumption on body weight are summarized in Table 4.

**TABLE 4:** Walnut consumption effects on body weight.

	Design of study	Part of plant	Dose	Finding	Reference
Clinical studies	Clinical trial *N* = 245 Overweight and obese women	Walnut	18% energy	↓Body weight ↓BMI	Le et al. (2016)
	Clinical trial *N* = 100 A standard diet with reduced energy density or an energy‐reduced diet enriched with walnuts (15% of energy)	Walnut	15% of energy	↓Body weight	Rock et al. (2017)
	Clinical trial *N* = 25	Walnut		↓Body weight	Olmedilla‐Alonso et al. (2008)
	Clinical trial *N* = 112 A diet with or without walnut	Walnut	56 g/day	No effect on anthropometric measures	Njike et al. (2015)
	Clinical trial *N* = 36	Walnut/black walnut	30 g/day	No significant effect on body weight	Fitschen et al. (2011)
	Clinical trial *N* = 46 Walnut‐enriched diet and diet without walnut	Walnut	56 g	No change in anthropometric measures	Katz et al. (2012)
	Clinical trial *N* = 20	Walnut	586 kcal/day	↑Level of satiety (3–4 days) Not detect effects on body weight	Brennan et al. (2010)
	Clinical trial *N* = 90	Walnut	12% of energy	No significant change in weight gain	Sabaté et al. (2005)
	Clinical trial *N* = 356	Walnut	28–56 g/day	No significant effects on body fat or body weight	Bitok et al. (2018)
In vitro & in vivo studies	In vivo	Walnut		Body weight in METS‐TG (a mouse model of MetS) is more than wild type mice	Scott et al. (2017)
	In vivo Alloxan‐induced diabetic rats	Walnut	21.3 g/42.6 g/85.2 g	↑Body weight	Onwuli et al. (2014)
	In vivo	Walnut oligopeptide solution		↑Muscle wasting due to aging (muscle mass and physical activity) Be effective in elderly individuals	Fan et al. (2022)
	In vivo Injection of streptozotocin (40 mg/kg) on days 6, 7, and 8 of pregnancy	Walnut (oil)	Appropriate dose: 900 mg/kg	↓Body weight	Sun et al. (2020)
	In vivo/in vitro	Walnut kernel	2.4 g	Body mass gain	Stanisic et al. (2021)

### 6.1 Effects of walnut kernel enriched diet on body weight

#### 6.1.1 Clinical studies

A randomized, crossover, placebo‐controlled study involving 25 volunteers demonstrated that consuming meat products with walnuts significantly reduced body weight compared to baseline measurements (Olmedilla‐Alonso et al., 2008). Additionally, the 1‐year behavioral weight loss intervention involving 245 overweight women with a walnut‐enriched diet resulted in reductions in body weight and BMI (Le et al., 2016). In another study with 100 participants, a walnut‐enriched reduced‐energy diet led to a significant 8.9% reduction in body weight (Rock et al., 2017).

Nijke and coworkers represented that daily consumption of walnuts had no significant effects on anthropometric measures in a clinical study involving 112 participants (Njike et al., 2015). Additionally, some studies have suggested that daily walnut consumption may lead to weight gain, although the increase was not found to be significant (Sabaté et al., 2005). In another randomized parallel trial conducted over 2 years on healthy elderly individuals, daily consumption of walnuts (28–56 g/day) did not show a significant impact on body weight or body fat (Bitok et al., 2018).

According to studies, daily consumption of walnut kernels appears to have conflicting effects on body weight. It seems that consuming walnut kernels has no significant impact on increasing or decreasing body weight. However, due to the promising effects of walnut consumption on other complications of MetS, walnuts can be added to a diet without concern about the negative effects on BMI or body weight.

**FIGURE 3:**
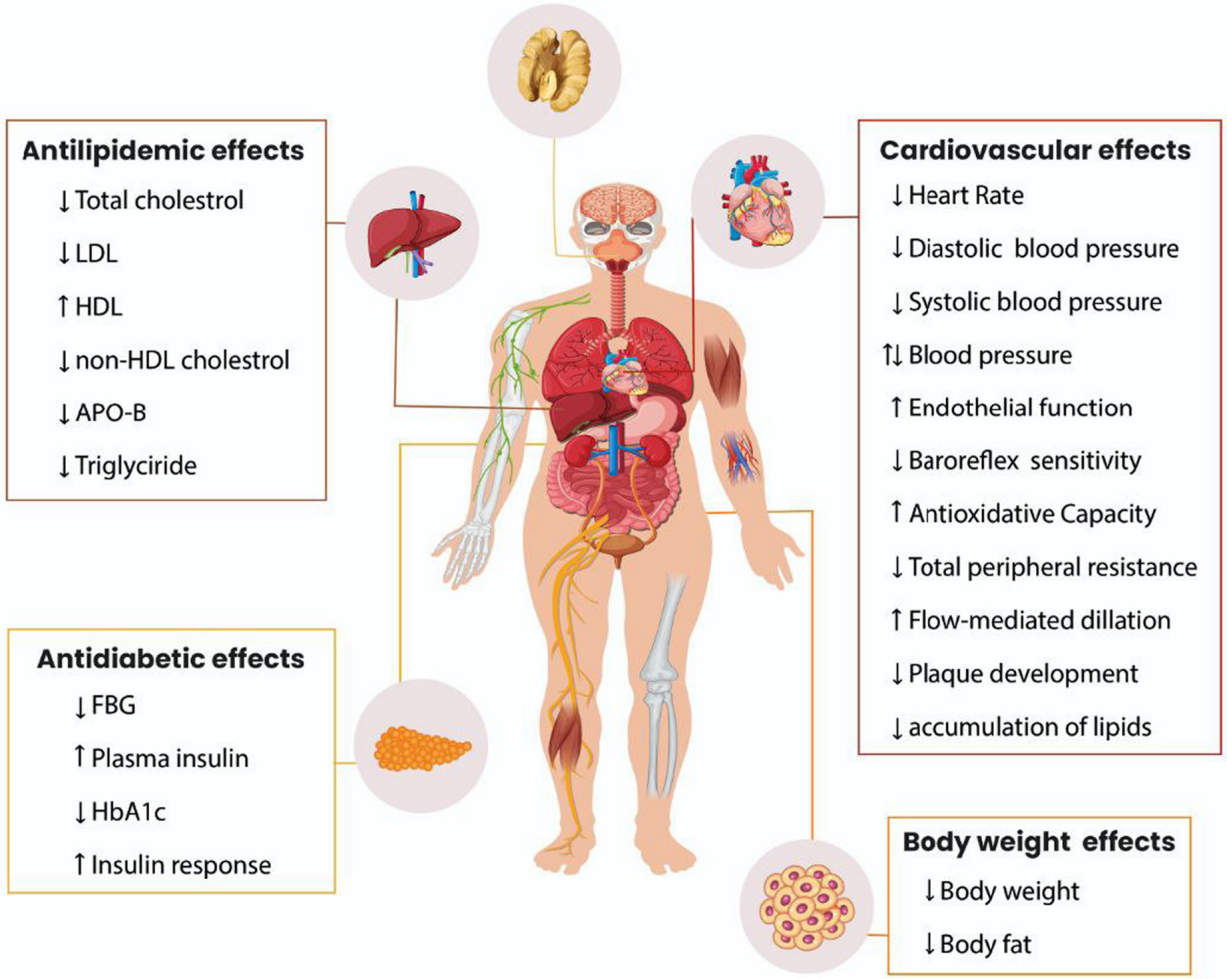
Different anti‐metabolic syndrome effects of the walnut kernel.

Ultimately, the therapeutic effects of consuming walnut kernels on MetS complications, including the reduction of heart rate, diastolic and systolic blood pressure, total lipid profile, diabetic complications, and body weight, are illustrated in Figure 3.

## 7 EFFECTS OF OTHER PARTS OF *J. REGIA* ON METS

In addition to the walnut kernel, other parts of *J. regia* such as leaves, green husk, septum, and hull have a desirable biological effect that is attributed to high amounts of phenolic compounds with potential antioxidant effects. Some of the phenolic compounds found in walnuts include juglone, ferulic acid, vanillic acid, ellagic acid, chlorogenic acid, and coumaric acid (Ebrahimi et al., 2017). The effect of these parts of *J. regia* on MetS was summarized in Table 5.

**TABLE 5:** The effect of other parts of *Juglans regia* on MetS.

	Design of study	Part of plant	Dose	Finding	Reference
Walnut leaves	Clinical trial *N* = 50	Leaf extract	200 mg/day	↓Systolic blood pressure ↓Body weight ↓BMI Without effects on blood glucose concentration in diabetic patients	Rabiei et al. (2018)
	Clinical trial *N* = 61	Leaf extract	100 mg/kg twice a day	↓Total cholesterol ↓Triglyceride levels ↓FBG ↓HbA1c	Hosseini, Huseini, et al. (2014) and Hosseini, Jamshidi, et al. (2014)
	Clinical trial *N* = 58	Leaves aqueous extract	200 mg/kg two times a day	↓Serum fasting HbA1C ↑Insulin level ↓Blood glucose level	Hosseini, Huseini, et al. (2014) and Hosseini, Jamshidi, et al. (2014)
	In vivo/in vitro Streptozotocin‐induced diabetic rats	Leaf powder	25, 50 and 100 mg/kg twice daily	+In vivo: revert hypercholesterolemia	Mollica et al. (2017)
	In vivo Streptozotocin nicotinamide ‐induced type 2 diabetes	Leaf	200 mg/kg	↑Fibronectin type III domain containing 5 (FNDC5) +Prevent weight loss in type2 diabetes ↓Fasting blood glucose, ↑Adiponectin	Atila Uslu and Uslu (2022)
	In vivo/In vitro Streptozotocin‐induced diabetic rats	Leaf powder	25, 50, 100 mg/kg twice daily	In vitro: inhibition of α‐glucosidase and α‐amylase In vivo: prevents hyperglycemia and multi‐organ failure associated with diabetes mellitus	Mollica et al. (2017)
	In vivo/In vitro	Leaves of walnut, olive, nettle and saltbush		Antioxidant α‐Glucosidase inhibition ↓Glucose absorption	Said et al. (2008)
	In vivo Alloxan/streptozotocin induced	Leaf	60 g/kg body weight/day	↓FBS ↑Granulated and normal β cells ↑The density of islets in the pancreas	Jelodar et al. (2007)
	In vivo Alloxan and streptozotocin‐induced diabetes in rats	Leaf extract	200 mg kg	↓FBS ↓HbA1c ↑β‐cells number ↑Insulin ↓Insulin resistance (by ↑ADP and ↑FNDC5, ↓FBG) ↑Insulin sensitivity ↓Reduced TNF‐α levels	Javidanpour et al. (2012)
	In vivo Streptozotocin‐induced diabetes	Leaf extract	200 mg/kg/day	Degeneration of sciatic nerves, increased caspase‐3, iNOS, and COX‐2 expression, lipid peroxidation, and nociceptive response ↓Blood sugar ↓Catalase activity and lipid peroxidation level ↓Blood glucose level The decadence of the retina attenuated	Nasiry, Khalatbary, and Ahmadvand (2017) and Nasiry, Khalatbary, Ahmadvand, et al. (2017)
	In vivo Streptozotocin‐induced	Leaf extract	200 mg/kg/day	↓Fasting blood sugar Prevention and treatment of diabetic nephropathy	Nasiry et al. (2019)
	In vivo Streptozotocin‐nicotinamide induced diabetic rats	Leaf extract		↓Serum glucose ↓HbA1c ↓Total cholesterol ↓Serum triglycerides	Mohammadi et al. (2012)
	In vivo Diabetes induced with alloxan	Ethanolic extracts of leaf	200 mg/kg	↓Fasting blood sugar ↑Insulin level ↓Glycosylated hemoglobin ↓Size of Langerhans	Asgary et al. (2008)
	In vitro	Leaf extract (methanolic)	25 μg/mL	↑Glucose uptake Inhibition of PTP1B	Pitschmann et al. (2014)
	In vitro	Leaf aqueous extracts		Inhibition of α‐amylase, which is time‐ and concentration‐dependent	Rahimzadeh et al. (2014)
Walnut septum	In vivo Streptozotocin induced diabetes	Walnut septum		Anti‐inflammatory and anti‐oxidant activity	Zhang et al. (2022)
	In vivo D‐galactose (D‐Gal)‐induced aging model and naturally aged rat	Walnut/walnut septum extract		↓Level of blood glucose Inhibition of acetylcholinesterase	Rusu, Georgiu, et al. (2020)
	In vivo Streptozotocin induced diabetes	Walnut septum		↓Insulin resistance ↑Lipid metabolism ↓Liver damage	Zhang et al. (2022)
	In vitro	Septum extract of walnut		+ Inhibition of α‐glucosidase and lipase, which may interfere with carbohydrate or fat metabolism in food +Antidiabetic effect	Rusu, Fizesan, et al. (2020)
Walnut green husk	In vivo High‐fat diet‐induced rats	Walnut green husk polysaccharides		Prevented abnormal changes in total cholesterol, triglyceride, NEFA levels, HDL‐C levels	Wang et al. (2021)
	In vivo	Walnut green husk		Prevention from inflammatory injury in the liver Inhibition of the release of inflammatory cytokines Activation of NF‐kappa B Production of endotoxin	Peng et al. (2015)
	In vivo High‐fat diet‐induced rats	Walnut green husk polysaccharides		↓Fat accumulation Prevents abnormal weight gain	Wang et al. (2021)
	In vivo High‐fat diet‐induced obesity	Walnut green husk		↓Body weight gain ↓Fat accumulation ↓Adipose tissue hypertrophy of high‐fat diet ↓Total cholesterol, ↓Total triglyceride, ↓LDL, ↑HDL	Wang et al. (2019)
	In vivo Alloxan and streptozotocin‐induced diabetes in rats	Walnut green husk extract	200 mg kg	↑β‐cells number ↑Insulin	Javidanpour et al. (2012)
Walnut bark	In vivo Tyloxapol model	Bark extract	100 mg/kg b.w.	↓Hepatic lipid peroxidation ↓Cholesterol levels	Almonte‐Flores et al. (2015)
	In vivo	Hydromethanolic extract of barks of *J. regia*	200 mg/kg	Anti‐diabetic effect similar to the use of glibenclamide	Benahmed et al. (2021)
	In vivo Diabetes induced with streptozotocin	Bark extract	200 mg/kg	↓Blood glucose	Almonte‐Flores et al. (2015)
	In vitro	Bark		α‐Glucosidase, α‐amylase, and β‐galactosidase inhibition	Boulfia et al. (2021)
	In vitro	Bark (Methanolic extract)		Inhibition of glycation, the percentage Inhibition of glycation was concentration‐dependent	Ahmad et al. (2012)

### 7.1 Walnut leaves

The walnut leaves are also used in traditional medicine. The extract of walnut leaves has shown important biological effects due to secondary metabolites, especially phenolic compounds including flavonoids and naphthoquinones (Hosseinzadeh et al., 2011).

Walnut leaves are a valuable source of polyphenolic compounds such as hydroquinone, 4‐hydroxybenzoic acid, quercetin derivatives, and caffeic acid. The extract of walnut has a high antioxidant capacity and may be introduced for the treatment of inflammatory‐related diseases (Altemimi et al., 2023).

In a double‐blind study with 50 diabetic patients, 200 mg/d of hydroalcoholic extract of walnut leaf significantly reduced the body weight (*p* = .028), BMI (*p* = .030), and SBP (*p* = .005) in comparison to baseline (Rabiei et al., 2018).

Clinical studies conducted with walnut leaves have demonstrated a significant reduction in FBS levels (*p* < .05) and HbA1C levels (0.9 mg/dL vs. baseline). Walnut leaves could significantly increase insulin levels by 1.4 in comparison to baseline (Hosseini, Huseini, et al., 2014; Hosseini, Jamshidi, et al., 2014).

In a study by Mollica et al. (2017), walnut leaf powder in streptozotocin‐induced diabetic rats indicated a relapse of hypercholesterolemia.

Nasiry, Khalatbary, and Ahmadvand (2017) and Nasiry, Khalatbary, Ahmadvand, et al. (2017) reported in an in vivo study that walnut leaf extract significantly reduced the FBS (*p* < .001). Mohammadi et al. (2012) suggested the walnut leaf has more vital antiglycation ability in diabetic test rats after consumption of 28 μg/mL walnut leaf extract. Another in vivo study by Jelodar et al. (2007) reported a significant increase in normal B cells, granular B cells, and Langerhans cells density upon consumption of walnut leaves.

In vitro studies have demonstrated the inhibition of α‐glucosidase and α‐amylase by walnut leaf extract (Mollica et al., 2017; Rahimzadeh et al., 2014). Pitschmann et al. (2014) demonstrated the increase in glucose uptake and PTP1B inhibitory effect of walnut leaves.

A study on walnut leaf extract in streptozotocin‐ and nicotinamide‐induced diabetes indicated prevention of weight loss in rats with type 2 diabetes (Atila Uslu & Uslu, 2022).

Overall, it appears that the oral consumption of walnut leaf extract may effectively control blood glucose levels and serve as a promising treatment for diabetes through various mechanisms, including α‐amylase inhibition, increased glucose uptake, and PTP1B inhibition.

### 7.2 Walnut septum

The walnut septum, the wooden membrane that separates the two halves of the walnut kernel, has demonstrated important biological effects both in vivo and in vitro, particularly in the treatment of diabetes. In a study on streptozotocin‐induced diabetic rats, the extract from the walnut septum reduced oxidative stress and inflammation (Zhang et al., 2022). Another study exploring the benefits of both walnut kernels and the septum in a D‐galactose‐induced aging model and naturally aged rats indicated potential hypoglycemic effects of the septum (Mateș et al., 2023).

In vitro experiments showed the inhibitory activity of walnut septum against α‐glucosidase and lipase. Additionally, walnut bark could inhibit α‐amylase, β‐galactosidase, and α‐glucosidase enzymes (Ahmad et al., 2012; Boulfia et al., 2021).

According to the literature, the antioxidant and anti‐inflammatory effects of walnut septum are related to phenolic compounds. These compounds interrupt the lipid peroxidation cycle and modulate the signaling pathways including PI3K/Akt/mTOR, Nrf2/EpRE, and NF‐kB (Mateș et al., 2023).

### 7.3 Walnut green husk

Another walnut by‐product is the green husk that covers the shell of mature walnut fruits. Despite being considered a waste product, this part of walnuts is used as an herbal medicine because of the presence of biological compounds.

An in vivo study conducted in diabetic rats supports the beneficial effects of the walnut green husk on increasing B‐cell count and insulin levels (Javidanpour et al., 2012).

Interestingly, different parts of the walnut yield distinct results. Walnut green husks have been shown to prevent abnormal weight gain by modulating lipid homeostasis (Wang et al., 2021) and reducing the body weight, fat accumulation, and hypertrophy of adipose tissue in cases of obesity due to high‐fat diet (Wang et al., 2019).

## 8 CONCLUSION

To address the prevalence of MetS, it is crucial to identify effective solutions with minimal adverse effects. This review article aims to explore the impact of *J. regia* on MetS. While many studies have emphasized investigating the potential benefits of consuming walnut kernels in reducing the risk of MetS, limited research has occurred on exploring the effects of other components of *J. regia* on MetS.

Despite numerous studies on the impact of consuming walnut kernels on MetS, it remains unclear whether these benefits are due to PUFA or secondary metabolites. Randomized trials have produced conflicting results regarding the effect of a diet enriched with walnut kernels on MetS. Overall, walnut kernels are edible nuts that are commonly included in daily diets. Numerous studies have shown that consuming walnuts and a diet enriched with n‐6 PUFA and n‐3 PUFA can have positive effects on MetS.

**FIGURE 4:**
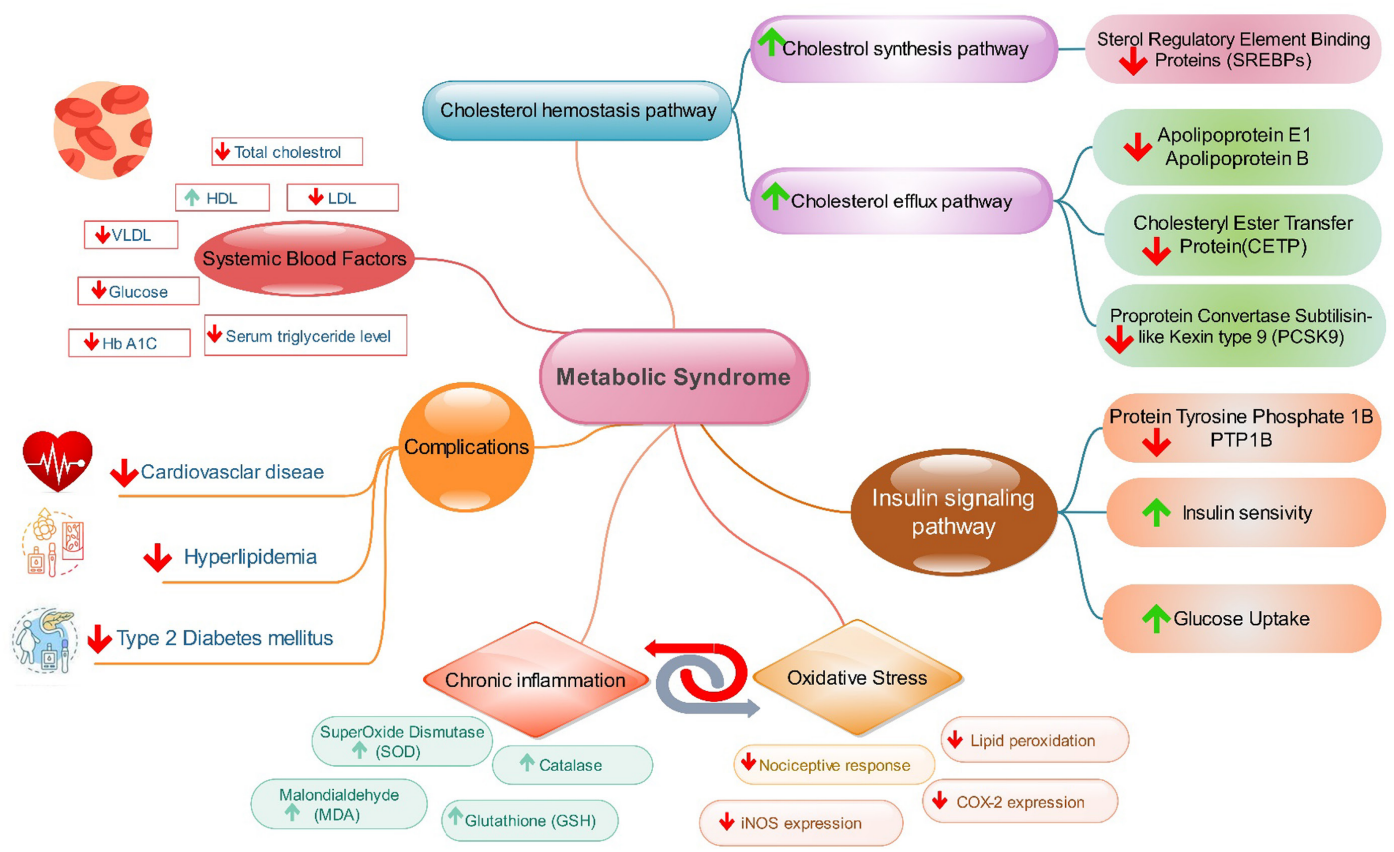
The signaling pathways involved in the therapeutic effects of *Juglans regia* on metabolic syndrome.

It appears that walnut kernel activates the cholesterol hemostasis pathway through SREBPs, PCSK9, and CETP. Additionally, modifications in insulin signaling pathways through protein tyrosine phosphatase 1B (PTP1B), inhibition of inflammatory response, and oxidative stress lead to a decrease in MetS complications. Figure 4 summarizes the pathways involved in the therapeutic effect of the walnut kernel on metabolic syndrome.

In conclusion, a diet enriched with walnut kernels can help monitor complications associated with MetS by reducing diastolic blood pressure, improving blood lipid profiles, lowering FBS levels, and increasing insulin sensitivity. Additionally, other by‐products of walnuts such as leaves and green husks have also shown to be effective in managing MetS. Walnut leaf extract has been found to have a potent antidiabetic effect through various mechanisms. Meanwhile, the green husk of walnuts has been shown to aid in weight loss and reduce fat accumulation. Overall, consuming walnuts can improve lipid profiles and decrease the risk of MetS complications.

## AUTHOR CONTRIBUTIONS

**Melika Samei:** Investigation (equal); writing – original draft (lead). **Nafiseh Dowlatkhahi:** Investigation (equal); writing – original draft (supporting). **Motahareh Boozari:** Investigation (equal); methodology (equal); supervision (equal); writing – review and editing (equal). **Hossein Hosseinzadeh:** Conceptualization (lead); methodology (equal); supervision (equal); writing – review and editing (equal).

## CONFLICT OF INTEREST STATEMENT

The authors declare no conflicts of interest.

## Data Availability

Data sharing does not apply to this article as no datasets were generated or analyzed during the current study.
